# 8^th^ International conference on management and rehabilitation of chronic respiratory failure: the long summaries – Part 3

**DOI:** 10.1186/s40248-015-0028-x

**Published:** 2015-10-06

**Authors:** Nicolino Ambrosino, Richard Casaburi, Alfredo Chetta, Enrico Clini, Claudio F. Donner, Michael Dreher, Roger Goldstein, Amal Jubran, Linda Nici, Caroline A. Owen, Carolyn Rochester, Martin J. Tobin, Guido Vagheggini, Michele Vitacca, Richard ZuWallack

**Affiliations:** Weaning and Pulmonary Rehabilitation Unit, Auxilium Vitae Rehabilitation Centre, Volterra, (PI) Italy; Los Angeles Biomedical Research Institute at Harbor-UCLA Medical Center Torrance, California, 90502 USA; Respiratory Diseases & Lung Function Unit, Department of Clinical & Experimental Medicine, University of Parma, Parma, Italy; Department of Medical and Surgical Sciences, University of Modena-Reggio Emilia, Reggio Emilia, Italy; Mondo Medico, Multidisciplinary and Rehabilitation Outpatient Clinic, Borgomanero, (No) Italy; Department of Cardiology, Pneumology, Angiology and Intensive Care Medicine, University Hospital Aachen, Aachen, Germany; West Park Healthcare Centre, University of Toronto, Toronto, Canada; Division of Pulmonary and Critical Care Medicine, Edward Hines Jr. Veterans Affairs Hospital and Loyola University of Chicago Stritch School of Medicine, Hines, IL 60141 USA; Pulmonary and Critical Care Section, Brown University, Providence Veterans Affairs Medical Center, Providence, RI USA; Division of Pulmonary and Critical Care Medicine, Brigham and Women’s Hospital/Harvard Medical School, Harvard Institutes of Medicine Building, Boston, MA 02115 USA; The Lovelace Respiratory Research Institute, Albuquerque, NM 87108 USA; Yale University School of Medicine, VA Connecticut Healthcare System, New Haven, CT USA; Respiratory Unit and Weaning Center, Salvatore Maugeri Foundation, IRCCS Institute of Lumezzane (BS), Lumezzane, Italy; Pulmonary and Critical Care, St Francis Hospital, Hartford, CT 06106 USA

**Keywords:** Ethics, Long-term ventilation, NIV, Palliative care, Telemonitoring, Weaning

## Abstract

This paper summarizes the Part 3 of the proceedings of the 8^th^ International Conference on Management and Rehabilitation of Chronic Respiratory Failure, held in Pescara, Italy, on 7 and 8 May, 2015. It summarizes the contributions from numerous experts in the field of chronic respiratory disease and chronic respiratory failure. The outline follows the temporal sequence of presentations.

This paper (Part 3) presents a section regarding Moving Across the Spectrum of Care for Long-Term Ventilation (Moving Across the Spectrum of Care for Long-Term Ventilation, New Indications for Non-Invasive Ventilation, Elective Ventilation in Respiratory Failure - Can you Prevent ICU Care in Patients with COPD?, Weaning in Long-Term Acute Care Hospitals in the United States, The Difficult-to-Wean Patient: Comprehensive management, Telemonitoring in Ventilator-Dependent Patients, Ethics and Palliative Care in Critically-Ill Respiratory Patients, and Ethics and Palliative Care in Ventilator-Dependent Patients).

## Background

This paper summarizes the Part 3 of the proceedings of the 8^th^ International Conference on Management and Rehabilitation of Chronic Respiratory Failure, held in Pescara, Italy on 7 and 8 May, 2015. It summarizes the contributions from numerous experts in the field of chronic respiratory disease and chronic respiratory failure. The outline follows the temporal sequence of presentations.

## Moving across the spectrum of care for long-term ventilation

### Rationale

As technology advances, therapeutic options for individuals with chronic respiratory failure requiring short- and long-term ventilator support increase. This section will review old and new indications for ventilator therapy, implementation and feasibility of these types of complex interventions, potential methods to improve their applicability and safety, and economic issues resulting from their use.

### Moving across the spectrum of long term ventilation (Roger Goldstein)

References to mechanical ventilation are found in the writings of Hippocrates (460–375 BC) and Paracelsus (1493–1541). However, since the 20^th^ century, long term mechanical ventilation (LTMV) has become important in the management of two overlapping groups of patients; those who have recovered from an acute episode of respiratory failure but who require ongoing ventilatory support despite being clinically stable and those who require ventilation electively to avoid the requirement for urgent ventilation. Added to the above, is the increased awareness of the value of a rehabilitative focus, to enhance function and improve autonomy of the ventilator assisted individual (VAI) in a non ICU environment.

#### Mandatory ventilation

**Fig. 1:**
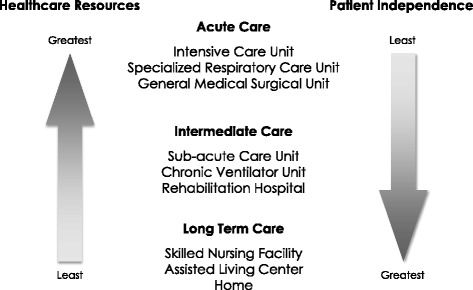
Potential sites of care for patients requiring PMV. Sites toward the bottom of the figure have fewer medical resources and lower costs but allow greater patient independence and a higher quality of life. PMV, prolonged mechanical ventilation

Patients receiving mandatory ventilation in the Intensive Care Unit (ICU) find themselves in an environment in which, understandably, the attending clinical team is focused on those with acute clinical issues. Their mobility is confined to the length of their ventilator tubing. Subsequent management outside the ICU will depend on the availability of resources such as a chronic assisted ventilator care unit, a long term acute care unit or a skilled nursing facility. The resource utilization is inversely related to the level of patient independence (Fig. 1) [1].

With the addition of rehabilitation many VAI are able to leave the ICU for an assisted living facility or even better, to go home, provided they have access to technical and clinical support services. Preparation for the relocation should begin in the ICU, as outlined in Table 1.

**Table 1:** Beginning rehabilitation in the ICU

• Change ventilator to a home friendly, portable, simple machine
• Change the tracheostomy tube to allow speech
• Teach the patient and family airway patency (suctioning) and manual ventilation
• Maximize mobilization (including outings)
• Introduce assistive technology for communication
• Assess swallowing
• Maximize nutrition
• Prepare to relocate

The availability of physical rehabilitation in the ICU has a positive influence on muscle function, independence and time to wean (Table 2) [2].

**Table 2:** Effects of physical training (6 weeks) for patients ventilated > 14 days (*n* = 39)

	Baseline		Sixth week	
	Control group	Treatment group	Control group	Treatment group
Shoulder flexors, kg	2.0	3.2	0.9	4.5^a^
Elbow flexors, kg	4.5	4.3	1.1	7.3^a^
Knee extensors, kg	4.1	4.1	1.8	7.3^a^
PImax -cmH_2_O	38.0	46.0	30.0	60.0^a^
PEmax cmH_2_O	42.0	45.0	35.0	62.0^a^
FIM				
ADL	6.0	6.0	6.0	13.0^a^
Mobility	5.0	5.0	5.0	9.0^a^
Executive	19.0	20.0	13.0	24.0^a^
Total	33.0	34.0	26.0	49.0^a^

8 treatment and 3 control subjects reached 12 h of ventilator free time

^a^ *p* < 0.05 compared with control

#### Elective ventilation

In contrast, the journey of elective ventilation often begins and ends at home. It hinges on the prompt initiation of elective ventilation for those whose conditions are progressing to cardio-respiratory failure. Good clinical and laboratory monitoring is important as the onset of respiratory failure may first be identified through a deterioration of nocturnal blood gases. Progression may include brief exacerbations with respiratory failure requiring ICU management and relatively easy weaning. As with mandatory ventilation, it is necessary to have access to home respiratory care services as well as scheduled monitoring after the initiation of ventilator support. The following example illustrates the relevance of monitoring in those likely to develop respiratory failure.

#### Case example

**Fig. 2:**
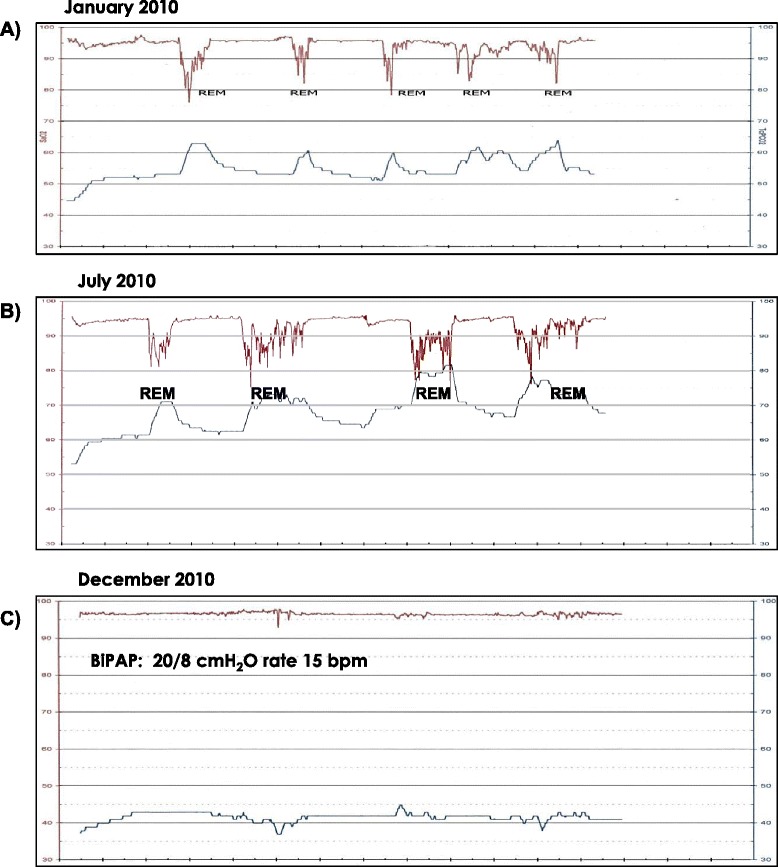
**a-c** Sleep study, two channel recordings. **a**) Initial study in a 45 year-old woman with thoracic restriction showing satisfactory oxygenation and mild nocturnal hypercapnia. **b**) Repeat evaluation after 6 months showing respiratory failure. **c**) After elective initiation of Bi-level Positive Airway Pressure Ventilation, showing stabilization of nighttime gas exchange which was associated in an improved clinical state

A 45 year-old woman with thoracic restriction developed gradually progressive dyspnea on exertion. Her vital capacity was 43 % predicted, her total lung capacity was 44 % predicted and her forced expired volume in one second to forced vital capacity was 90 %. Arterial blood gases taken on room air showed her to have: pH 7.39, PaCO_2_ 46 mmHg, PaO_2_ 78 mmHg, SaO_2_ 95 %. After an episode of pneumonia a two channel overnight recording showed satisfactory oxygenation, mild nocturnal hypercapnia with periodic (likely REM related) worsening of gas exchange (Fig. 2a). She began to feel unwell over the next few months and on repeat evaluation after 6 months (Fig. 2b) she was noted to have marked hypercapnia. Bi-level positive airway pressure ventilation was initiated electively (Fig. 2c) and her clinical state as well as her blood gases stabilized. She remains stable on nocturnal non- invasive positive pressure ventilation.

#### Prevalence of HMV

**Fig. 3:**
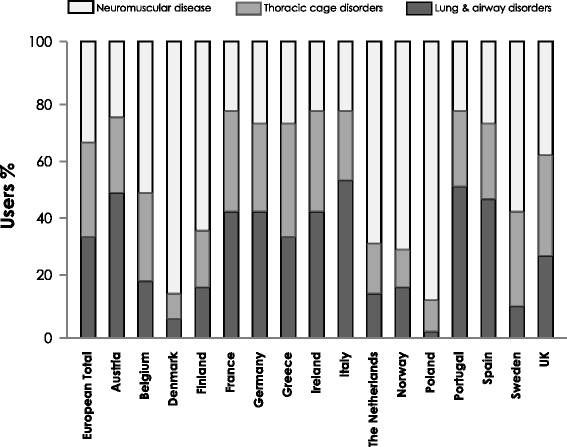
HMV in Europe by Category: Percentage of users in each disease category by country: From [4] with permission of the European Respiratory Society.  neuromuscular;  thoracic cage disorders;  lung and airway disorders. HMV was defined as a period of ventilation ≥ 3 months outside of an acute care hospital

The prevalence of home ventilation is influenced by the increasing incidence of the underlying disorders, the increased knowledge of the healthcare providers (HCP) regarding the option of being safely ventilated outside of the ICU and the guidelines and recommendations of professional societies regarding LTMV [3]. It is also influenced by the attitudes and preferences of the patient and family as well as the availability of formal and informal (caregiver) support services. In Europe (Fig. 3) [4] the prevalence of HMV varies widely (France 17/100,000 to Poland 0.1 per 100,000), as does the distribution of diseases requiring ventilatory support (thoracic cage disorders, neuromuscular disorders and airway disorders).

If the patient is unable to return home immediately, a chronic assisted ventilatory care (CAVC) unit will provide a safe, non-acute care environment with a rehabilitative focus, to optimize health related quality of life and promote autonomy. The CAVC unit requires a multi-dimensional continuum of services, by an interdisciplinary team trained both in ventilator management and rehabilitation. The preferred patient is medically stable, mentally alert, understands that ventilatory assistance is long term, is prepared to participate in comprehensive training and will relocate with appropriate supports. In order for a patient to return home, it must be safe and have the required utilities as well as trained care givers. The availability of home health care, technical support and organized follow-up is critical.

#### Ventilator-assisted individuals’ perspectives

User perspectives [5] suggest that irrespective of ventilation being elective or mandatory, the most difficult period for coping is the initial 3 months after returning home. When asked to give their experience of LTMV, ventilator-assisted individuals voiced both positive and negative experiences regarding mobility, symptoms, equipment concerns and social implications. Disappointingly, not all users felt that they had made an informed choice when they started ventilation or when it became permanent.

Ventilator-assisted individuals (VAIs) have noted the relevance of both physical and psychological adjustments to being ventilated [6]. They describe the positive impact that their physicians’ confidence in the effectiveness of LTV has on them as well as the importance of the opinion of other VAIs. The adjustment to LTMV is more difficult when it is initiated in the ICU especially if impaired verbal communication limits their decision to initiate ventilation. The following quotes are illustrative of some of the experiences of the ventilator-assisted individuals:

#### Adjustment quotes

“As I became stronger I thought what is so different about my care that I could not learn?”

“Lots to do with cleaning equipment and tracking supplies but I do it as part of my daily routine and its easier.”

“I wish I would have started with the ventilator earlier as I might have avoided getting so sick and the ICU experience.”

#### Satisfaction and success quotes

“My energy was back, I was renewed, I felt wonderful. It was noisy as the air escaped with every breath but that didn’t bother me as I was so glad to have this thing to help me breathe.”

“It just gave me so much more energy to work through the day. I figured what the heck, why struggle when I don’t have to.”

Their overall recommendations included:

1. Enhancing quality of life through improvements in healthcare funding, healthcare professional education and ventilator equipment design.
2. Reducing barriers through improved public education, access to government programs and community accessibility.
3. Educating prospective users about LTV, accepting it as soon as it is indicated and being aware of the personal responsibilities involved.
4. Engaging HCP to support the users’ decision for HMV, improving HCP education regarding HMV
5. Partnering with an experienced VAI whose expertise can be of great value in establishing optimum care.

#### Caregiver burden

Separate from the paid caregivers, informal caregivers, usually family members, are essential to the development of an environment that enables the ventilator-assisted individual to live safely at home. These informal caregivers often underestimate the care burden involved, which is especially high when that individual also has neuromuscular disease (NMD). Semi-structured caregiver interviews [7] of those looking after patients with NMD highlighted their sense of duty and their huge commitment. However caregiver burnout was evident and the need for professional support, especially in the initial weeks of their loved ones returning home, was evident.

The following quotes are illustrative of some of the issues that the caregivers face:

#### Restriction in day-to-day life

“I am a prisoner in my own home, at my own will. Although I don’t regret it, this is the way I feel.”

“But it’s being deprived of my life, my family, my future, of my expectations for my retirement, of everything – it’s gone.”

#### Physical and emotional burden

“There are things that are hard for me to help him as my hands get stiff. I have a herniated disc too. I think it is all from him.”

“So even at night when you sleep, you are sleeping with one eye open. I’m a very light sleeper and I’ve been having a problem for many years because of a lack of sleep.”

“I have depression and I feel sad most of the time. Sometimes I hate my life.”

#### Training and education

“It was very hard to come home the first time after the hospital. Even though we got trained you don’t know what to expect so it was very difficult.”

“It’s not enough to only teach the medical things, you need to know what to expect in the long run. Knowing about the disease really helps.”

“It’s quite overwhelming in the beginning.”

#### Tele-medicine follow-up

Key points that contribute to caregiver success are summarized in Table 3. Regular pre-scheduled follow-up, the ability for VAI initiated medical support and respite care for the ventilator-assisted individual or caregiver, are especially important.

**Table 3:** Key points for caregiver success

• Huge commitment made by family caregivers
• Caregiver burden despite formal home ventilation training
• The need to introduce post training discussion, assessment of caregiver skills
• Increase professional supports in the initial weeks
• Increased home follow-up to recognize issues
• Closer integration between hospital and community
• Respite care for caregivers

The frequency and complexity of follow up is determined by both medical and social factors. It will vary among individuals and in the same individual at different points in time. The arrival of modern telemedicine technology has resulted in more frequent home based rather than the institutional follow-up. For example, video-conferencing is achievable with a personal laptop computer, linked to healthcare professionals through videoconferencing software and high speed internet. Regular sessions can be scheduled for the VAIs convenience. The patient, family, caregiver and health team can all be present as can a pulmonologist and a community care access case manager. This approach has the advantage of enabling more frequent follow-up at home and broad health team access. It is also less expensive than home visits.

#### Summary

Although the spectrum of long term ventilation begins with either mandatory or elective ventilation, the ideal destination is home or if this is not possible a safe non acute care facility with a multidisciplinary team trained in both LTMV and rehabilitation. User perspectives emphasize that the most difficult period of coping is the first few months after returning home, when both physical and psychological adjustments are necessary. Caregiver burden is substantial and under-recognized both by the healthcare team and by the caregivers when they make their initial commitment to accept a ventilator-assisted individual at home. Access to home healthcare and technical services is critical to successful home ventilation. Telemedicine technology using personal computer video-conferencing software has enabled more frequent, less expensive follow-up with improved access by the patient and the caregiver to healthcare professionals.

### New indications for non-invasive ventilation (Nicolino Ambrosino)

#### Key points

- The use of non invasive ventilation (NIV) is an option in acute hypercapnic respiratory failure, cardiogenic pulmonary oedema, acute respiratory distress syndrome (ARDS), community-acquired pneumonia, and weaning failure
- Evidence supports NIV during complicated bronchoscopy, some cases of transoesophageal echocardiography, and in some interventional cardiology
- NIV can reduce the need for deep sedation or general anaesthesia
- NIV should be considered with caution in severe communicable airborne infections likely to progress to ARDS
- The role of assisted ventilation during exercise training is still controversial
- NIV should be applied under close monitoring, and endotracheal intubation should be promptly available in the case of failure.
- A trained team, careful patient selection and optimal choice of devices, can optimize outcome of NIV

Non invasive ventilation (NIV) may be considered as one of the most important advances in respiratory medicine over the past 20 years, [8, 9] and is increasingly being utilized world-wide [10]. A PubMed search from January 1966 to March 2015 with the term “non invasive ventilation” offers 6904 papers. The International Consensus Conference 2001 [11] defines NIV as “any form of ventilatory support applied without endotracheal intubation (ETI)”. There is strong evidence (Level A) for the use of NIV to prevent ETI in acute on chronic respiratory failure, acute cardiogenic pulmonary oedema, and to facilitate extubation in patients with acute exacerbations of chronic obstructive pulmonary disease (COPD). Less evidence supports the use of NIV for patients with severe acute asthma exacerbations, post-operative or post-extubation acute respiratory failure (ARF), pneumonia, or acute respiratory distress syndrome (ARDS) [8, 9]. Nevertheless, many other potential applications have been proposed [12]. This review will focus on potential new indications for NIV.

Although potentially risky, bronchoscopy may be required for some severely hypoxaemic patients [13]. In the past, the American Thoracic Society (ATS) did not recommend flexible bronchoscopy and bronchoalveolar lavage (BAL) in such conditions when supplemental oxygen cannot correct an arterial oxygen tension (PaO_2_) at least to 75 mmHg or an arterial oxygen saturation (SaO_2_) to 90 % [14]. On the other hand, non-use of bronchoscopy in these high risk patients may result in less effective, empiric treatment. Until recently, when bronchoscopy wws needed in hypoxaemic conditions, only ETI and mechanical ventilation were available to provide adequate ventilation and oxygenation. Unfortunately, invasive mechanical ventilation is associated with complications related to ETI, baro- or volutrauma, and the loss of airway defense mechanisms. NIV has the potential to avoid these complications while ensuring a similar level of ventilatory efficacy and control of hypoxemia.

In a randomised controlled trial (RCT), mask Continuous Positive Airway Pressure (CPAP) reduced the risk of acute respiratory failure complicating bronchoscopy in severely hypoxaemic patients [15]. Another RCT in hypoxaemic patients showed that during bronchoscopy NIV increased the PaO_2_/inspiratory oxygen fraction (FIO_2_) ratio, whereas the patients randomised to only oxygen therapy showed a worsening in oxygenation [16]. NIV during bronchoscopy is also useful in hypercapnic COPD patients with pneumonia [17]. CPAP was able to reverse reductions in tidal volume and respiratory flow associated to flexible bronchoscopy in spontaneously breathing young children [18]. In patients with acute exacerbation of COPD due to community-acquired pneumonia, in danger of ETI and unable to clear secretions, NIV with early therapeutic bronchoscopy was feasible, safe and effective [19]. A recent study suggests that in awake, critically ill patients with moderate to severe hypoxaemia undergoing bronchoscopy, the application of NIV is superior to High Flow Nasal Cannula Oxygen in oxygenation before, during and after the procedure [20]. NIV during bronchoscopy may be performed by means of commercial or modified oronasal or full-face masks [21]. These reports support the use of NIV during fiberoptic bronchoscopy especially when risks of ETI are high, such as in immunocompromised patients. However, an expert team with skills in both endoscopy and NIV should be available for any emergency [12]. In general, this should be performed in ICU.

#### Transoesophageal echocardiography and interventional cardiology

In orthopnoeic cardiac patients needing transoesophageal echocardiography (TEE), NIV can reduce the need for deep sedation or general anaesthesia. NIV allows performance of continuous TEE examination in lightly sedated patients, avoiding ETI and general anaesthesia. The level of evidence is lower than in fiberoptic bronchoscopy and is more linked to author’s experience [22]. The author of this review is not aware of recommendations in such situations.

Recent advances in interventional techniques have made it possible to offer minimally invasive treatment of aortic valve stenosis to elderly or complex patients unable to undergo standard surgical procedures due to a compromised health status or severe comorbidities, such as pulmonary diseases [22]. Furthermore, orthopnoea may make it difficult for patients to stay supine. Our initial experience with NIV in interventional cardiology to support patients with severe pulmonary disease needing percutaneous implantation of an aortic bioprosthesis for severe valve stenosis was positive [23]. NIV reduced the need for general anaesthesia, relieved orthopnoea and prevented post-operative ARF [23]. As for TEE, the evidence behind this review is based mainly on author’s experience, and a large clinical trail would be needed to confirm this preliminary observation.

#### Interventional pulmonology

Intermittent Negative Pressure Ventilation (INPV) through a poncho-wrap may be useful in reducing apnoeas during laser therapy under general anaesthesia, thus reducing hypercapnia, related acidosis, and required oxygen supplementation with related explosion hazard [20]. Furthermore, compared with spontaneous ventilation, INPV in paralysed patients during interventional rigid bronchoscopy may reduce need of opioids, shorten recovery time, prevent respiratory acidosis, need for manually assisted ventilation, reduce the oxygen need and allow optimal surgical conditions [24, 25]. This author is aware that INPV is not commonly used in this condition, mainly due to lack of large randomized controlled trials. Accordingly, a review such this is important to disseminate experience and promote research in this area.

Video-assisted thoracoscopic surgery is a minimally invasive technique allowing for intrathoracic surgery without any formal thoracotomy and related complications [26]. We successfully used face mask NIV with regional anaesthesia during this technique which requires the exclusion of a lung from ventilation [27].

#### High transmissible infections

There are still insufficient data on the use of NIV during pulmonary infections, including pandemic respiratory infections [28]. NIV was used in patients with Severe Acute Respiratory Syndrome (SARS) in 2002–2003 and also during the H1N1 epidemic in 2009. Thereafter NIV has been used to treat ARF due to other infectious diseases, like pandemic avian influenza (H5N1). However, NIV in these conditions requires caution. Although studies of NIV use in ARF during H1N1 influenza [28, 29] do not report disease transmission from patients to healthcare workers, the World Health Organization (WHO) has included NIV among aerosol-generating procedures with possible risk of pathogen transmission [30].

The members of an International NIV Network examined the literature of NIV in SARS, H1N1 and tuberculosis. The conclusion was that early application of NIV in selected patients can reverse ARF. Furthermore there were only a few reports of infectious disease transmission among healthcare workers [31].

Despite these positive results, the guidelines from the European Respiratory Society (ERS)/European Society of Intensive Care Medicine (ESICM), WHO, the UK National Health Service, Hong Kong Lung Foundation and the American Association for Respiratory Care (AARC), suggest that NIV should not to be used as first-line therapy in H1N1-associated ARF for several reasons: [32, 33] 1) Poor clinical efficacy in severe ARF rapidly progressing to refractory hypoxaemia and ARDS; 2) More prevalent hypoxaemic instead of hypercapnic ARF in patients with H1N1; 3) Concern about aerosol droplet particle dispersion and spread of infection.

Technical issues in ARF caused by airborne infectious diseases include: 1) Ventilators with a double-line circuit without an expiratory port (like whisper, plateau exhalation valve, anti-rebreathing valve etc.) should be preferred. This can reduce the risk of dispersion of exhaled infected particles through the intentional leaks of a single line circuit; 2) Well customized face masks should be preferred to nasal masks to avoid the potential spreading of contaminated air particles from the mouth; 3) Healthcare workers should be aware of the potential risks of using NIV in such conditions taking appropriate precautions especially during the patient disconnection from the NIV; [34] 4) In general, patient isolation and protective measures also for care-givers should limit if not avoid disease transmission; 5) The use of other techniques such as high flow nasal cannula are controversial.

#### Palliative and end-of-life care

Most end-stage patients with chronic respiratory failure complain of dyspnoea in the last 3 months of life [35]. Breathlessness is often more severe in these patients than in those with advanced lung cancer [36]. As a consequence, NIV is being increasingly used to relieve dyspnea in these patients [37, 38]. Recent guidelines state the following: “As relief of dyspnoea with NIV may not relate to changes in arterial blood gases, it is appropriate to reassess the br0eathlessness experienced by patients receiving such ventilatory support at frequent intervals” [39]. Observational studies as well as clinical trials have recently confirmed the role of NIV in patients with chronic disease and poor life expectancy (with or without COPD), showing that this ventilatory technique may favourably reduce dyspnoea shortly after initiation, even without an associated episode of hypercapnic ARF [40]. About half of the patients survived the episode of respiratory distress and were discharged from the hospital.

A Task Force of the Society of Critical Care Medicine defined the approach to NIV use for end-stage patients who choose to forego ETI [41]. The use of NIV for patients with ARF could be classified into three categories: 1) NIV as life support with no preset limitations on life sustaining treatments; 2) NIV as life support when patients and families have decided to forego ETI; and 3) NIV as a palliative measure when patients and families have chosen to forego all life support, receiving comfort measures only. NIV should be applied after careful discussion of the goals of care, with explicit parameters for success and failure, by experienced personnel and in appropriate healthcare settings [41, 42]. The use of NIV in these circumstances should take into account ethical, legal and religious issues.

### Elective ventilation in respiratory failure - can you prevent ICU care in patients with COPD? (Michael Dreher, Michele Vitacca, Nicolino Ambrosino)

#### Key points

- Chronic respiratory failure is very frequently the final stage of the natural history of chronic obstructive pulmonary disease.
- The role of long-term non invasive positive pressure ventilation in improving survival in COPD patients with CRF is still discussed.
- Long-term night non invasive ventilation in these patients has some physiological and clinical benefits.
- Long-term non invasive ventilation should be reserved to individual patients.

Chronic respiratory failure (CRF) is very frequent in the end stage of the natural history of chronic obstructive pulmonary disease (COPD). Among other factors, inspiratory muscle dysfunction due to pulmonary hyperinflation may lead to ineffective alveolar ventilation resulting in chronic hypercapnia. Whether chronic hypercapnia is adversely associated with overall prognosis is still discussed, at least in patients on long term oxygen therapy (LTOT) [43].

Home long-term non-invasive positive pressure ventilation (NPPV) is widely used around Europe to treat CRF due to different aetiologies such as restrictive thoracic (RTD) and neuromuscular disorders (NMD), obesity hypoventilation syndrome and COPD [4].

The hypothesized - but not proven - mechanisms of action of long-term NPPV in stable hypercapnic COPD patients include: reverting hypoventilation; respiratory muscle unloading; resetting of respiratory centers; and cardiovascular effects. These mechanisms may work alone or in combination.

#### Hypoventilation

Physiological studies demonstrate that in these patients, NPPV is able to improve alveolar ventilation by increasing the tidal volume and reducing the respiratory rate [44].

#### Respiratory muscles

Inspiratory support is able to unload the inspiratory muscles, and positive end expiratory pressure (PEEP) counteracts the intrinsic PEEP associated with hyperinflation, [45] an effect more evident in acute exacerbations.

#### Respiratory centers

Compared with LTOT alone, the addition of night NPPV results in significant increases in day-time arterial oxygen (PaO_2_) and reductions of carbon dioxide (PaCO_2_) tension, total sleep time, sleep efficiency, and overnight PaCO_2_. Additionally, health-related quality of life with LTOT plus NPPV was significantly better than with LTOT alone. The degree of improvement in day-time PaCO_2_ correlates significantly with the improvement in mean overnight PaCO_2_ [46].

#### Cardiovascular effects

Nighttime NPPV may improve heart rate variability, reduce circulating natriuretic peptide levels, and increase the functional performance of patients with advanced but stable COPD - suggesting that night NPPV may reduce the impact of cardiac comorbidities in COPD patients [47].

#### Clinical results

Although home NPPV is widely accepted for the treatment of chronic hypercapnia due to respiratory or neuromuscular disease, whether stable hypercapnic COPD patients should routinely be offered this therapy is still discussed [48]. Recently, the role of ventilator management on physiological parameters and outcome in stable hypercapnic COPD patients has become more evident. It is suggested that its benefits depend on the ability of NPPV to substantially reduce PaCO_2_ through using “high” inflation pressures [49]. This was confirmed by prospective trials, showing an advantage of high over lower inspiratory pressure levels, with regard to improvements of lung function, blood gases, exercise-induced dyspnoea and health status [50, 51]. A multicenter study showed a highly significant survival advantage of NPPV (compared with standard care) when it was targeted to maximize hypercapnia reduction [52]. The findings of that study may influence the attitude of clinicians on the use of NPPV in patients with stable hypercapnic COPD. However, the effect of elective home NPPV on exacerbation frequency in stable hypercapnic COPD remains to be determined.

The use of NPPV is a first line treatment of acute on chronic hypercapnic respiratory failure in COPD patients [8]. However, once acute hypercapnic respiratory failure is successfully managed and these patients are discharged, there is an 80 % re-hospitalization rate due to another acute exacerbation over the following year [53]. Furthermore, long-term survival in this patient cohort remains poor [54]. Three relatively small studies investigated the effect of home NPPV after acute hypercapnic respiratory failure successfully treated in COPD patients. One study showed that, compared to sham (continuous positive airway pressure) ventilation, NPPV significantly reduced the probability of recurrent acute hypercapnic respiratory failure [55]. Another study compared home NPPV versus standard therapy in chronic hypercapnic respiratory failure patients after acute exacerbation in order to prevent clinical worsening [56]. The authors demonstrated that the probability of clinical worsening was significantly lower in the group receiving home NPPV, with additional improvements observed in exercise capacity. The third, retrospective, study demonstrated better survival in COPD patients discharged after acute respiratory failure with home NPPV compared to those discharged without this form of therapy [57].

#### Pro/Con long-term NPPV

There is limited evidence to support the provision of NPPV in the home environment after successful treatment of acute hypercapnic respiratory failure in COPD patients. However, those studies supporting this intervention had limitations, including small sample size, retrospective nature, and a lack of control group. Struik et al. [58] evaluated whether home NPPV after successfully treated acute respiratory failure reduces re-hospitalization and improves survival. The investigators randomized patients to home NPPV or standard treatment 48 h after “acute” ventilator support was terminated. The study failed to show a positive effect of home NPPV on time to readmission or death. This was not anticipated and stands in clear contrast to the smaller studies published before. Looking deeper into the study it can be seen that both groups had reductions in PaCO_2_ over time. Therefore, one explanation of why this multicenter study was negative was the fact that patients were randomized too early: given the natural course of the disease, patients might have been randomized while they were still recovering from acute hypercapnia. Therefore, home NPPV might have been prescribed to patients not suffering from chronic hypercapnia. This study underscores the importance of carefully selecting patients for home NPPV.

Another study [59] was unable to show an improvement in 2-year survival, despite the demonstration of reductions in day-time PaCO_2_ (while breathing oxygen), improvements in health status, and reductions in readmissions. Therefore, it appears unlikely that differences in 1 year survival between the Köhnlein study [52] and others [58, 59] are due only to “high inspiratory pressures” or simply reductions in PaCO_2_ [52]. As a matter of fact, the control group of the Köhnlein study suffered from a high mortality rate, which may indicate that severity of disease rather than the correction of hypercapnia or the beneficial effect of “high inspiratory pressures” primarily drives survival in patients treated with NPPV. Furthermore, claim that chronic hypercapnia is associated to worse survival is questionable - at least in those patients receiving long-term oxygen therapy [43]. Furthermore there is growing evidence that mortality in COPD is influenced by several other factors, such as exercise capacity, comorbidities and inflammatory status [60].

Overall, home NPPV has been shown to improve important physiological parameters in stable hypercapnic COPD patients by the use of a treatment strategy which sufficiently decreases elevated PaCO_2_ levels [51]. By doing so, long term survival can be significantly improved. However, the influence of home NPPV to prevent re-hospitalization is still unclear, and future trials are needed to identify the subgroup of COPD patients which benefits most from home NPPV. From a clinical point of view, it seems reasonable that patients with acute hypercapnic respiratory failure needing mechanical ventilation in hospital and suffering from prolonged hypercapnia, the ones you can define as acute on chronic hypercapnia respiratory failure, might benefit most. However, inconclusive data are available up to date and further investigation is needed in this area.

## Conclusion

There is conflicting evidence regarding the effect of NPPV on reducing health care utilization and mortality in acute on chronic respiratory failure due to COPD. We need to better assess when to initiate this therapy in patients with hypercapnia in this setting. Once stable hypercapnia is proven, NPPV may improve survival and health status. Therefore, despite recent studies adding some new data, the authors cannot recommend the widespread use of this therapeutic intervention after an episode of acute-on-chronic respiratory failure in COPD. There is simply not enough evidence to support it. Instead, this modality should be reserved for individual cases, treated in specialized centers experienced with NPPV for the treatment of stable hypercapnic COPD.

### Weaning in long-term acute care hospitals in the United States Hospitals (Martin Tobin, Amal Jubran)

The non-intuitive term “long-term acute care hospital (LTACH)” is viewed as the antonym of short-term acute care hospital (STACH). The term originates with Medicare bureaucrats who define LTACH as an acute care hospital with a mean length of stay of at least 25 days. Prolonged ventilation has been variously defined, as greater than 2 days, 14 days or 29 days, and now is generally, but arbitrarily, defined as at least 21 consecutive days of mechanical ventilation [61]. A number of different names has been applied to facilities focused on weaning from prolonged ventilation, including step-down units, respiratory intensive care units, and intermediate care units, which are located within a short-term acute care hospital, or a LTACH, which commonly is a free-standing hospital [61].

Much of the driving force behind LTACHs relates to money. Costs for ICU beds in the US have increased dramatically: by 30.4 % per day between 2000 and 2005 [62]. Costs for mechanical ventilation in the US are estimated at $27 billion, representing 12 % of all hospital costs. Because of the formula employed for payment by Medicare, the diagnosis-related group (DRG) system, hospitals begin to lose large amounts of money when length of stay exceeds 14 days. Transfer of patients out of an acute ICU to a step-down unit or LTACH saves money on a per-day basis, largely by lower nurse-to-patient ratios, and by increase in the availability of ICU beds for more profitable cases such as elective surgeries.

Although money is the dynamo behind the expansion of LTACHs, it is also recognized that patients being weaned from prolonged ventilation have different needs than patients in acute ICUs. These patients require a greater rehabilitative, as opposed to life-support, focus and they may benefit from being transferred out of the high technology environment of an ICU.

Given the colossal sums of money spent on caring for patients requiring prolonged mechanical ventilation, it is amazing that these patients have attracted minimal attention from science-oriented investigators as opposed to health economists. This review is focused on science and on how best to wean patients receiving prolonged ventilation rather than on the economics of ventilator care.

Between 2000 and 2010, Jubran et al. conducted a randomized controlled trial to determine whether the method selected for weaning influenced weaning duration in patients receiving prolonged ventilation [63]. The two arms of the study consisted of pressure support and trials of unassisted breathing using an O_2_ delivery device connected to a tracheostomy tube (a trach collar). The primary aim of the study was to determine the length of time required for weaning with pressure support versus trach collar. Patients were eligible for entry into the study if they had received mechanical ventilation for at least 21 days. All patients underwent a screening procedure, which consisted of breathing unassisted through a trach collar for 5 days.

One hundred and sixty patients did not develop distress during the five days and were considered to have been successfully weaned and were not randomized. Three hundred and sixteen patients developed respiratory distress during the 5-day period and were judged to have failed the screening procedure and were randomized to wean with pressure support or trach collar. Patients randomized to trach collar were disconnected from the ventilator and allowed to breathe through the tracheostomy. During the first day, the patient was allowed to breathe unassisted for a maximum of 12 h. The patient was then reconnected to the ventilator and assist-control ventilation was instituted for the next 12 h. On the second day, the 12-h trach-collar challenge followed by assist-control ventilation was repeated. On the third day, the patient was disconnected from the ventilator and allowed to breathe unassisted through the trach collar up to 24 h. In the pressure-support arm, on the first day the initial level was titrated to achieve a total respiratory frequency of less than 30 breaths per minute. Attempts were made to decrease pressure support by 2 cmH_2_O three times each day. When a patient was able to tolerate pressure support of no more than 6 cmH_2_O for at least 12 h, the ventilator was disconnected and the patient allowed to breathe unassisted through the tracheostomy up to a maximum of 24 h each day.

The primary outcome, weaning duration, defined from the first day of randomization to the day the patient was successfully weaned, was shorter with trach collar than with pressure support: 15 versus 19 days. Patients were considered weaning successes when they breathed without ventilator assistance for at least 5 days. A Cox proportional hazards model revealed that the rate of successful weaning was 1.43 times faster with trach collar than with pressure support. Mortality was equivalent in the two arms, but, of course, the study was not powered to detect a difference in mortality. Of the entire 500 randomized and non-randomized patients, 54 % were alive at 6 months after enrollment and 45 % were alive at 12 months. This survival rate is surprisingly high. To put the numbers in perspective, 1-year survival in older (66 years) patients ventilated in an ICU was approximately 40 % [64, 65]. That is, the LTACH patients in the study of Jubran et al., who were ventilated for 67 days, had a 1-year mortality comparable to ICU patients who were ventilated for 9 days. Indeed, 72 % of the 260 patients who had been weaned by discharge were alive at 12 months.

What explains the faster pace of weaning with a trach collar than with pressure support? One explanation lies with how doctors make decisions. During a trach-collar challenge, the amount of respiratory work is determined solely by the patient – the ventilator cannot do any work. As such, a physician observing a patient breathe through a trach collar has a completely clear view of the patient's respiratory capabilities. During pressure support weaning, a clinician's ability to judge weanability is clouded because the patient is receiving ventilator assistance and it is extremely difficult to distinguish between how much work the patient is doing and how much work the ventilator is doing [66]. Accordingly, clinicians are more likely to accelerate the weaning process in patients who perform unexpectedly well during a trach-collar challenge than when a low level of pressure support is being used. This notion is borne out by the Kaplan-Meier plot, which shows that the superiority of trach collar over pressure support was evident within the first ten days of the study [63].

In summary, the number of patients requiring prolonged mechanical ventilation, whether they are placed in a short-term acute care hospital or some other location, is likely to increase enormously in the next few decades. The use of a trach collar accelerates the pace of weaning of such patients by more than 40 % as compared to weaning using pressure support.

### The difficult-to-wean patient: comprehensive management (Guido Vagheggini, Nicolino Ambrosino)

#### Key points

- Prolonged weaning is defined as the need for more than three weaning trial failures, or 7 days from the first spontaneous breathing trial
- Specialized weaning units allow greater weaning rate, better functional status
- Survivors may suffer from long-lasting physical and cognitive disabilities resulting in impaired quality of life
- Physiotherapy is part of the comprehensive management
- Protocol-based weaning strategies may be effective
- High risk of dysphagia has been reported in critically ill patients.

Prolonged weaning is defined as the need for more than three weaning trial failures, or 7 days from the first spontaneous breathing trial [67]. It occurs in up to 14 % of patients admitted to intensive care units (ICU) and treated with invasive mechanical ventilation, accounting up to 37 % of ICU costs [68, 69]. These patients have a hospital mortality up to 32 %, [70] and fewer than half of them survive beyond 1 year [71]. Specialized weaning units allow better results, in terms of percentage of patients free from mechanical ventilation, and functional status at discharge, particularly if the organizational model is focused to the early post-acute period [72].

Clinical outcomes of critically ill patients admitted to ICUs showed a huge improvement in the last decades, due to the advancements in critical care. Nonetheless, survivors may suffer from long-lasting physical and cognitive disabilities resulting in impaired quality of life, even after long time from the acute illness [73].

It has been reported that muscle wasting in critically ill patients starts in the very first week of illness being more severe in patients with multiorgan failure than in those with a single organ failure [74]. Physiotherapy must be considered as an integral part of the comprehensive management of these critically ill patients. A strategy of early comprehensive rehabilitation based on interruption of sedation and physical and occupational therapy is safe and well tolerated, resulting in better functional outcomes at hospital discharge, shorter delirium, and more ventilator-free days [75]. Current guidelines and recommendations promote early mobilization in ICU, to reduce deconditioning and other immobility related complications, and increase functional independence and psychological well being [76]. Neuromuscular electrical stimulation (NMES), able to exercise muscles with minor burden on cardio-ventilatory system, can be easily performed in the ICU and applied to muscles of patients laying in bed to prevent the ICU neuromyopathy [77].

Despite no definitive results exist regarding the application of a fixed protocol-based procedure to discontinue mechanical ventilation, the use of this care plane has proven to be effective when applied to the weaning process in the critical care area [78]. Recent advances in mechanical ventilation (NAVA, closed loop) were developed to facilitate weaning in acute care and in prolonged weaning [79]. Some recent meta-analysis [80] showed that weaning with closed-loop ventilators significantly decreased weaning time in critically ill patients, however, its utility when compared with respiratory physiotherapist protocolized weaning is still a matter of debate [81].

Aside from regaining respiratory autonomy and clinical stability, the removal of tracheotomy may represent a difficult challenge in prolonged weaning patients, and currently available recommendations are still largely based on subjective criteria rather than on standardized protocols. High risk of dysphagia has been reported in critically ill patients, and an accurate evaluation of swallowing disorders may reduce risk of infections and failure of tracheostomy weaning [82].

### Telemonitoring in ventilator dependent patients (Michele Vitacca)

#### Key points

- Home mechanical ventilators may be equipped with remote monitoring tools in order to improve physician supervision, with the aim to adapt settings to the needs and comfort of the patient
- Economic, regulatory and legal impacts of home telemonitoring will be important in its adaption by health care systems
- Relevant issues are prescription criteria, modalities of follow-up, team expertise, technologies, adherence, bundling of services, and outcomes

#### Introduction and rationale

Patients with chronic respiratory insufficiency requiring home mechanical ventilation (HMV) have a high, although underestimated prevalence in Europe [4]. Home mechanical ventilation requires patient and family cooperation, nevertheless clinical conditions, technology needs, lack of professional supervision, and acute exacerbations make its management a difficult task [4, 83]. Provision and maintenance is often carried out by external companies, without any accepted standardisation, and a regular feedback to the clinical centres is usually lacking [84]. The need to reduce healthcare costs has prompted the development of telemedicine for home assistance [85]. However, only few controlled studies evaluating its effectiveness are available so far. Identification and selection of HMV patients who may benefit from such tele-monitoring approach represent key factors [86]. There are real challenges when providing HMV, including patient and caregiver training, adequacy of respiratory care, and reimbursement.

The aim of a recent ERS Task Force has been to develop and establish a European network of clinical experts in HMV for a critical analysis of the current status of tele-monitoring services in ventilator-dependent patients and provide a consensus document on common clinical criteria, equipment, and facilities.

#### Overview on telemedicine, telemonitoring definitions

Telemedicine (TM) is the distribution of health services - in conditions where distance is a critical factor - by health care providers using information and communication technologies to facilitate the exchange of important clinical information [86]. TM dimensions may be divided into functionality, applications and technology categories. Functionality, in turn, may be divided into:

- Tele-consultation: Second opinion on demand between patient/family and staff or among health operators; opinions, advice provided at distance between two or more parties separated geographically
- Decision support system: Alerting health personnel, in response to a sentinel value, who then contact the patient or caregivers
- Remote diagnosis: Identifying a disease by the assessment of the data transmitted to the receiving party through instrumentation monitoring a patient away.
- Tele-therapy: Direct prescript**i**on
- Mentoring (i.e., tele-coaching): Direct reinforcement or recorded messages/communications to improve adherence
- Telemonitoring: Digital/broadband/satellite/wireless or bluetooth transmission of physiologic and other non-invasive data (i.e. biological storage data transfer)
- Tele-evaluation: On-demand data transfer to use as biological outcome measures
- Telecare: Network of health and social services in a specific area; in case of emergency, patient calls medical personnel, emergency call service or members of family
- Telerehabilitation: The system which allows for receiving home care and guidance on the process of rehabilitation through connections for point-to-point video conferencing between a central control unit and a patient at home.
- Emergency calls: Helpline service that gives the ability to initiate a call for help to an Operation Centre, usually active 24 h a day throughout the year
- Teleconference-Audio: Electronic two-way voice communication between two or more people located in different places, which make use of transmission systems voice, video and/or data.
- Telepresence: Use of robotic devices and other devices allowing to perform a task in a remote place by manipulating instruments and receiving sensory information and reactions.
- Telespirometry: Remote control of a flow volume curve through a spirometer which is then sent to a central processing and reporting

#### Indications for TM in ventilator-dependent patients

In general, TM would be appropriate in patients receiving supported ventilation outside an acute care hospital, including those receiving non invasive ventilation (NIV) and those receiving invasive ventilation (IV). The latter would include those with as weaning failure and those undergoing some kind of a weaning process.

Telemonitoring could be used for:

Ventilator Weaning: As an adjunct to weaning outside the acute care hospital [87].

ALS (amyotrophic lateral sclerosis) [87–89]: TM in ventilated patients due to ALS has been addressed in the medical literature. One study by De Almeida and colleagues demonstrated that the device was user-friendly [89]. A prospective, single blinded, controlled trial of TM versus no TM in 40 ALS showed that telemonitoring reduced health care utilization and probably had beneficial effects on survival and functional status [87]. TM is cost-effective in these patients representing major cost savings to the NHS in the order of 700 euros/patient/year.

Chronic respiratory failure [90–100]: In general, TM for chronic respiratory failure is feasible, tends to reduce hospitalisations, relapses, and urgent GP calls, helps facilitate titration of oxygen, and helps with changes in mechanical ventilation settings.

#### Equipment/technology available

The components of the technological dimension can be grouped into three sets, of variables: synchronicity, network design, and connectivity [85]:

- *Synchronicity* is used here to incorporate both timing and technology.
- *Network design/configuration* includes three modalities: Virtual Private Networks, the open internet, and social networks, in which information is posted and shared.
- *Connectivity*, wired and wireless, provides different levels of bandwidth and the attendant speed and resolution or quality of service.

A wide range of remote health monitoring systems is available. The correct level of technology should be: i) safe; ii) feasible; iii) effective; iv) sustainable; v) and flexible to meet different patient’s conditions and needs.

#### Time of TM follow up

Tele-monitoring has been proposed for home use, with a proposed time of use ranging from 3 months to 4 years [87, 89, 90, 101–106].

#### Legal issues

The use of TM has highlighted several medico-legal issues that must be addressed as this intervention achieves greater acceptance [107]. Further governmental, ethical, legal, regulatory, technical, and administrative standards for remote medicine will be necessary to assist individuals and organizations in providing safe and effective services.

#### Economical considerations

As awareness of the potential role of at-home telecare and telemonitoring in the care of ventilator-dependent (VD) patients increases, potential roadblocks also become more apparent. This type of care is labor-intensive and costly, [107] and the current medical literature on its cost/effectiveness presents contrasting results [89, 95, 100, 104].

Analyses comparing institutional versus at-home interventions in VD patients focused on traditional outcomes such as hospitalization rates. This narrow approach ignored such important methodologies and outcome areas as:

- Telemonitoring *vs.* formal caregiver monitoring in at home VD patients’ care, in order to potential savings of telemonitoring compared to high intensity labor home activities;
- Quality of life comparison between the above two groups.

To evaluate the real cost/effectiveness of a new method such as remote monitoring in this population, it is important to understand what “standard therapy” and “usual therapy” actually refer to in published papers. Often the comparator treatment is quite variable among European countries. “Standard therapy” can be considered to encompass the drug prescription, control by the general practitioner (GP), structured outpatient programs, and pathways of integrated on-demand home visits with dedicated paths in highly disabled patients. Each of these programs may have different indications, applicability and costs, making generalizations from comparison s with a new protocol of remote monitoring problematic. Despite preliminary studies that have shown an advantage in applying telehealth systems, more recent research casts some doubts on their superiority with regard to effectiveness or cost savings.

#### Tele-rehabilitation for home mechanical ventilated patients

Integrating telehealth into existing health service delivery patterns will require a reliable technological infrastructure, effective clinical demonstrations, assessment of practitioners' readiness, and careful integration of technologies into workflow and policy synchronization. Future initiatives will cover developing organizational models, promoting sustainability and participation, creating feasible, economical, effective and safe technological models, developing new technical devices and software and – ultimately - demonstrating effectiveness at the clinical level (including cost reduction, enhancement of quality of life, and patient/caregiver support).

#### Role of telemedicine in sleep-related breathing disorders

Sleep-related breathing disorders (SRDB) are a group of pathologies characterized by abnormalities of the respiratory pattern during sleep. The two most important are obstructive sleep apnea (OSA) and the reduction of ventilation during the night (hypoventilation syndromes). Recent investigations have evaluated the application of telemedicine in the diagnosis, treatment and compliance in OSA patients. For hypoventilation syndromes, the following areas should be considered in investigations. a) Indications for treatment; b) NIV titration; c) Optimal NIV devices and quality control; d) Follow-up strategies; e) Procedures to obtain adequate ventilation; and f) Treatment adherence. Finally, cost-effectiveness must ultimately be addressed.

#### Telemedicine at the end of life

Telehospice, the use of telemedicine technologies to provide services to hospice patients [108–110], may offer an innovative solution to the challenges of providing high-quality, cost-effective end-of-life care.

#### Future considerations

Tele-monitoring could become be a key element (part of the ‘total package’) in the integrated management of the patient requiring home mechanical ventilation for chronic respiratory failure. Future outcome assessment could include:

1. Physiological and functional impact: Impact of home mechanical ventilation on physiologic and functional status variables.
2. Survival and quality of life: Outcomes related to quantity and quality of life; including daily living activities, social interactions, autonomy, self-management, etc.
3. Health services: Use of health services directly related to home mechanical ventilation: phone calls, technical home visits.
4. Resources use: Use of health services (emergency visits, admissions, out-patient visits…) and, specially, the caregiver burden.

### Ethics and palliative care in critically-ill respiratory patients (Michele Vitacca)

#### Key points

- The trajectory of the dying process in COPD patients is highly variable
- Lack of surveillance and inadequate services with absence of palliative care is a routinely experience.
- Patients with COPD most frequently request information on the diagnosis and disease process, its treatments, prognosis, maintaining quality of life, and advance care planning.
- All too often, palliative home care programs and hospice admissions for end of life care in respiratory patients are insufficient or absent.

In the USA, non-oncological respiratory causes account for 8 % of all deaths and 9.6 % of deaths in individuals over age 65 years; of these 56 % are from COPD [111]. The COPD time course is characterized by a progressive worsening of dyspnoea, reduced effort tolerance, and more frequent exacerbations and hospitalizations [36]. In COPD, oxygen therapy and mechanical ventilation (MV) improve survival and morbidity in acute-on-chronic respiratory failure [36]. The downhill trajectory in COPD patients is variable and not as predictable as that of other chronic diseases [36]. In general, the course of COPD is that of progressive long-term disability, with periodic exacerbations and unpredictable timing of death which characterize dying with chronic multiorgan failure [36]. In a provocative paper, Curtis et al. [36] proposed that, after a serious analysis of the conditions and clinical status, we would define a patient needing palliative care when he/she has a low chance of recovery, poor rehabilitation potential, and high organizational complexity and instrumental requirements.

Multiple factors influence quality of care for COPD patients requiring palliative care. Three examples include: 1) the presence of anxiety and depression, which are as common in advanced COPD; 2) the use of advance care planning; and 3) effective communication among the patient, family and health care providers.

Those individuals in their last days of life (typically, an estimated of death within the next 7 days) may be defined as end of life (EOL) patients. Caring for these patients should be defined as potentially "futile", i.e. disproportionate measures in terms of quality and quantity of care with poor expected quality of life.

The hospital is often the location where EOL decisions are made for patients with end-stage COPD [112]. The patient, family and health care providers are usually involved in this process; all provide different perspectives and expectations. In a recent survey Nava et al. [113] showed that, in European respiratory intermediate care units and high dependency units, an EOL decision was made in 21.5 % of patients. Withholding of treatment, do-not-intubate/do-not-resuscitate orders, and noninvasive mechanical ventilation (NMV) as the ventilatory care ceiling are the most common forms of decision-making. In the same survey, the investigators showed that competent patients, together with nurses, are often major players in EOL decisions. A common notion is that European intensive care unit (ICU) physicians, in most cases, do not experience difficulties with EOL decisions. However, Sprung et al. [114] underline that EOL decisions change according to diagnosis, countries and doctors’ religion. Another important point is the well-known difficulty in accurately predicting outcomes (including death) for COPD patients admitted the ICU.

Wildman and colleagues [115] investigated whether clinicians' prognoses matched survival outcome in patients hospitalized in 92 ICU and three respiratory high-dependency units in the United Kingdom with severe acute exacerbations of COPD. Of this group, 517 (62 %) survived out to 180 days. In general, the clinicians' prognoses were too pessimistic: their predicted survival was 49 %. Furthermore, for their patients with what they considered the severest disease, predicted survival was 10 %, but in reality it was 40 %.

Gerstel at al. [116] pointed out that one of the main problems is that withdrawal of life support in the ICU is often a complex process, influenced strongly by patient and family characteristics. In this study, in almost one-half of the group, the decision to withdraw life-sustaining therapy took longer than one day. Those patients with longer decision-making were younger, had a longer length of stay in the ICU, received more life-sustaining interventions, were less likely to have a diagnosis of cancer, and had more decision-makers involved in the process. A longer decision process leading to withdrawal of life support was associated with increased family satisfaction, as was extubation before death.

As compared to hospitalized patients with lung cancer, individuals with COPD were more likely to receive mechanical ventilation, tube feeding, and resuscitation [117]. Furthermore, in COPD patients, mechanical ventilation had greater short term effectiveness, based on survival to hospital discharge (76 % vs. 38 %), and had higher 2-month and 6-month survival. Curtis and colleagues [118] pointed out an additional important problem related to EOL is the strategy of communication: the physicians’ frequent difficulties in discussing EOL care with patients and their families and caregivers

Health care utilization is strongly weighted toward the end of life in COPD as well as in other diseases. For example, Andersson and colleagues [119] showed that more than 68 % of all COPD admissions and 74 % of all days in hospital occurred in the 3.5 years before death. The last 6 months of life accounted for 22 % and 28 % of all COPD admissions and days, respectively. Suboptimal surveillance, inadequate services, and absence of palliative home care are common in severe COPD patients with EOL issues [120]. This also holds for respiratory patients who are housebound with high levels of morbidity and high requirements for community health services. COPD patients approaching EOL require, at a minimum, education on diagnosis and disease process, available treatment modalities, what they have to do and what to expect, and information on prognosis. Despite this, only 32 % of respiratory patients report discussing EOL cares with their physicians [120]. Stated barriers in this study included, “I would rather concentrate on staying alive than talk about death” or “I’m not sure which doctor will be taking care of me if I get very sick.” Thus, it is necessary to identify areas of communication that physicians do not address and areas that patients rate poorly, including talking about prognosis, dying and spirituality.

Finally, issues in COPD patients with mechanical ventilation (MV) deserve mention. Marchese et al. [121] describe survival, predictors of long-term outcome and attitudes in patients treated at home by tracheostomy-intermittent positive-pressure ventilation (TIPPV) over a 10-year period. Sixty-four out of 77 patients (83 %) were pleased to have chosen MV with tracheostomy and 69 (90 %) would choose this option again. Forty-two caregivers (55 %) were pleased the patients had chosen home mechanical ventilation (HMV), but 29 (38 %) reported major burdens. TIPPV is generally well-received by patients, is considered safe, and often permits survival for relatively long periods of time.

Vitacca et al. [35] describe the family’s perception of care delivered to home MV patients during the last 3 months of life. Eleven Respiratory Units submitted a binary 35-item questionnaire with 6 domains (symptoms, awareness of disease, family burden, dying, medical troubles and technical problems) to close relatives of 168 deceased patients (41 % with COPD). The majority had prominent respiratory symptoms and were aware of the severity and prognosis of their disease. Family burden was high, especially with respect to financial burden. During hospitalisation, 74.4 % of patients had been admitted to an ICU and 27 % received resuscitation manoeuvres. Hospitalisations and family financial burden were unrelated to diagnosis and use of MV, and families of the patients did not report major technical problems regarding the use of ventilators [35]. Steele et al. [122] describe how hospice care can offer expertise for palliation and may be used as a bridge between hospital and home.

Communication with patients and families about EOL issues is an important component of proper medical care that is often neglected in the training of clinicians. Although direct studies of health care provider interaction in COPD in this setting are not readily available, Vitacca et al. [123] showed how to communicate bad news to caregivers of patients with amyotrophic lateral sclerosis (ALS). In particular, caregivers require major assistance during the delicate times of discussing advance care planning and directives and critical treatments decisions. Clinicians are, therefore, an important target group for education on this type of communication. The Calgary–Cambridge model for medical consultations and the SPIKES (Setting up, Perception, Invitation, Knowledge, Emotions, Strategy and Summary) [124] for breaking “bad news” provides examples of consultation guides that integrate patient agenda with biomedical issues. Communication of advance care planning, defined as an ongoing discussion among patients and family members may be a more effective mean to meet patients' wishes.

Future direction for non-oncological respiratory patients with severe disease or EOL issues will focus on outcomes as well as skills and interventions for doctors, nurses and respiratory therapists. Many questions remain unanswered: Which are the important, measurable prognostic indicators? Which are indicators for unmet patient and caregiver needs? Which interventions optimize quality of life in this setting? Which are the important, relevant and priority criteria for palliative network and hospice access? What are the economic and social costs? What about enterable nutrition withdrawing? What about bio-ethical issues? What about patient information as awareness and self decision?

In conclusion, for respiratory patients with EOL issues we need to:

- Offer the best practice to ameliorate the pervasive effects of the disease
- Recognition that medication alone is insufficient to achieve optimal outcome
- Control the often overwhelming symptoms (such as dyspnoea) and psychological symptoms
- Focus our care on our patient and the family
- Allow for and foster a continuous presence of family, friends and religious assistance
- Give time and place to our patient to say everyone “good bye”
- Talk to our patients and relatives using their language
- Listen to our patients and their families and caregivers
- Consider patients’ preferences
- Not unduly prolong suffering to maintain life in some EOL situations
- Consider hospice and “palliative care” as opportunities for our patients

### Ethics and palliative care in ventilator dependent patients (Guido Vagheggini, Nicolino Ambrosino)

#### Key points

- The care of end-stage patients requires a progressive reduction of useless and “futile” treatments and an increasing approach to relieve of symptoms
- Clinicians are involved in surrogate or joint decision making
- Patient centered supportive care should respect patients’ values and preferences
- Physicians and healthcare professionals are challenged by prognostic accuracy of patient survival.
- End-stage COPD patients receive far less opiates than cancer subjects to alleviate dyspnea.

The care of end-stage patients requires a progressive reduction of useless and “futile” treatments and an increasing approach aimed to prevention and relieve of symptoms, including maintenance and improvement of quality of life of patients and families. Nevertheless end-stage lung diseases like Chronic Obstructive Pulmonary Disease (COPD) have a similar severe prognosis as lung cancer, but lower risk of Intensive Care Unit (ICU) admission refusal compared to cancer or haematological malignancies patients [125, 126].

Due to the different evolution of end stage chronic respiratory diseases, and to the lack of physician’s accuracy in judgment of prognosis in terminally ill patients, it may often be difficult to decide when to start palliative treatment [36, 127]. In these patients, the transition from the usual to the palliative and end of life care cannot be a step down, but should start simultaneously to the care, as soon as needed, and last until and after the death to ensure appropriate support to the family.

In these patients the main questions a clinician should face are: What might be desirable in terms of medical intervention? Should we pursue aggressive treatment or comfort treatment alone? In these patients, determining whether a patient is dying or not has become as important as the management of organ support therapy itself, as withdrawal or withholding of artificial life support may be determinant for their survival [128]. Very often clinicians are involved in surrogate or joint decision making, even in major medical decisions, so they need to be in partnership and communicating with surrogate decisions makers [129].

Patient centered supportive care should respect patients’ values and preferences, be coordinated and integrated in the care programme, and include an adequate information, communication and education. Physical comfort and emotional support of the patient should be pursued, with the involvement of family and friends, in order to share decisions and avoid abandonment experiences when care is redirected toward a more palliative purpose [130].

Physicians and healthcare professionals are challenged by prognostic accuracy of patient survival in patients with severe end-stage of COPD, and they are less likely to engage in end-of-life care planning in contrast with terminal diseases like cancer [131]. Home mechanical ventilation (HMV) is a growing issue in developed western countries; it is often administered as a life-sustaining treatment, but may have an important role also as a palliative treatment of dyspnea [4, 132, 133].

More recent guidelines include the use of pharmacological treatment of dyspnoea. Nevertheless, despite the beneficial response and the safety of opiates in end stage lung disease has been demonstrated, the end-stage COPD patients receive far less opiates than cancer subjects to alleviate dyspnea [134–136]. Conversely, end- stage COPD patients undergo more hospital and ICU admissions than cancer patients, an evidence that some issues in general and medical culture prevent appropriate supportive and palliative care in non-cancer end-stage lung disease [137]. Also in amyothrophic lateral sclerosis (ALS) patients, a high rate of hospital death is reported, the place of death depending widely on the attitude of the hospital and resources availability of the environment, more than on patient’s and families’ preferences [138].

In conclusion, in the care of end-stage lung disease patients, we must facilitate care in accordance with patient’s wishes, when possible, by exploring advance directives and involving family and care team in the development of the management plan. Continued and intensive efforts have to be addressed to palliate symptoms as earlier as possible during the clinical course of the illness, simultaneously to the care of treatable conditions, and recognizing the need of end-of-life care when appropriate.

In this perspective, harmonising acute care criteria for admission to ICU and criteria for long-term care is a crucial challenge to make more ethical the care provided to the patient.
